# Naturally occurring small molecules with dual effect upon inflammatory signaling pathways and endoplasmic reticulum stress response

**DOI:** 10.1007/s13105-024-01014-1

**Published:** 2024-03-19

**Authors:** Daniela Correia da Silva, Patrícia Valentão, David M. Pereira

**Affiliations:** REQUIMTE/LAQV, Laboratório de Farmacognosia, Departamento de Química, Faculdade de Farmácia, Universidade Do Porto, Rua de Jorge Viterbo Ferreira, Nº 228, 4050-213 Porto, Portugal

**Keywords:** IL-6, TNF-α, IL-1β, ATF4, CHOP, Caspase-1

## Abstract

The endoplasmic reticulum (ER) is determinant to maintain cellular proteostasis. Upon unresolved ER stress, this organelle activates the unfolded protein response (UPR). Sustained UPR activates is known to occur in inflammatory processes, deeming the ER a potential molecular target for the treatment of inflammation. This work characterizes the inflammatory/UPR-related molecular machinery modulated by an in-house library of natural products, aiming to pave the way for the development of new selective drugs that act upon the ER to counter inflammation-related chronic diseases. Starting from a library of 134 compounds of natural occurrence, mostly occurring in medicinal plants, nontoxic molecules were screened for their inhibitory capacity against LPS-induced nuclear factor kappa B (NF-κB) activation in a luciferase-based reporter gene assay. Since several natural products inhibited NF-κB expression in THP-1 macrophages, their effect on reactive oxygen species (ROS) production and inflammasome activation was assessed, as well as their transcriptional outcome regarding ER stress. The bioactivities of several natural products are described herein for the first time. We report the anti-inflammatory potential of guaiazulene and describe 5-deoxykaempferol as a novel inhibitor of inflammasome activation. Furthermore, we describe the dual potential of 5-deoxykaempferol, berberine, guaiazulene, luteolin-4’-*O*-glucoside, myricetin, quercetagetin and sennoside B to modulate inflammatory signaling ER stress. Our results show that natural products are promising molecules for the discovery and pharmaceutical development of chemical entities able to modulate the inflammatory response, as well as proteostasis and the UPR.

## Introduction

The endoplasmic reticulum (ER) is responsible for most of the protein production of eukaryotic cells, and thus cells rely on tight proteostasis regulation to survive. A set of signaling pathways has evolved in this organelle, rendering it able to detect and respond to anomalies in protein folding. As a whole, these signaling pathways are known as the unfolded protein response (UPR) [9, 30].

Three major signaling branches constitute the UPR network, each possessing its respective transmembrane sensor. All of these sensors are bound to chaperones, the most expressive being the binding immunoglobulin protein (BiP or GRP78). BiP binds misfolded proteins on the ER lumen on its C-terminal domain, releasing the aforementioned transmembrane sensors from its N-terminal domain and initiating the UPR. These transmembrane proteins are i) protein kinase RNA-like endoplasmic reticulum kinase (PERK), ii) inositol-requiring enzyme 1 (IRE1), and iii) activating transcription factor 6 (ATF6). The signaling cascades they initiate scale down global protein synthesis rates, while increasing the expression of selected genes and proteins that are involved in the expansion of the ER machinery or the UPR, ER-associated degradation (ERAD) or ultimately involved in cell death [3, 9, 19].

ER stress signaling pathway activation has been shown to occur in the pathogenesis of several types of diseases, being virtually omnipresent in chronic and age-related ones [4, 30, 57]. ER stress and the UPR are implicated in the pancreatic β-cell dysfunction underlying types I and II diabetes mellitus, viral infection, in proteinopathy-associated neurodegenerative diseases, and in the death or survival of cancer cells [19, 30].

Inflammatory signaling is traditionally associated to infectious diseases and mechanical trauma. However, not unlike ER stress, inflammation is deeply associated to chronic diseases, such as cancer, diabetes and neurodegenerative disorders [23]. The most broadly reported mechanism connecting ER stress to inflammatory signaling pathways is related to the fact that the UPR both reduces the synthesis and promotes degradation of several short-lived proteins, including the nuclear factor kappa B (NF-κB) inhibitor (IκB). Its eventual depletion owing to its relatively short half-life, contributes to the activation of the NF-κB [28, 41]. As so, ER stress and the resulting UPR directly potentiate the propagation of inflammatory processes. Furthermore, the UPR additionally results in the upregulation of several proinflammatory proteins. For instance, in intestinal cells ATF6 increases the expression of TNF-α and other inflammatory cytokines, leading to the hypothesis that inhibiting the activation of this signaling pathway may be useful to counter inflammatory bowel diseases [45].

Increased levels of the C/EBP homologous protein (CHOP), a UPR-associated transcription factor, induces the expression of IL-23, while ATF6 induces the transcription of acute phase proteins [14]. We have shown before that treating M1 macrophages (pro-inflammatory phenotype) with marine-derived molecules, such as ergosta-7,22,dien-3-ol results in downregulation of CHOP and also cyclooxygenase-2 (COX-2) and inducible nitric oxide synthase (iNOS), simultaneously resulting in the upregulation of NF-κB inhibitor IκB-α [40].

Previous works have shown that many described ER stress modulators are naturally-occurring small molecules [10, 41, 42]. In this work, we compiled and assessed over 100 small molecules, aiming to repurpose previously characterized and commercially available natural products. We investigated if these molecules could exert inhibitory effects on both inflammatory and ER stress signaling on THP-1 macrophages, thus providing additional advantages versus single-target molecules.

## Materials and methods

### Chemical library and reagents

Our chemical library consisted solely on commercially acquired natural products. (-)-Norepinephrine, ( ±)-dihydrokaempferol, 1,4-naphtoquinone, 18α-glycyrrhetinic acid, 3,4-dihydroxybenzoic acid, 3,4-dimethoxycinnamic acid, 3-hydroxybenzoic acid, 4-hydroxybenzoic acid, 5-methoxypsoralen, ajmalicine, berberine, betanin, betulin, boldine, catechol, cholesta-3,5-diene, chrysin, cynarin, daidzein, emodin, galanthamine, genistein, guaiaverin, hesperetin, hydroquinone, lupeol, myristic acid, naringenin, naringin, *p*-coumaric acid, phloridzin, pinocembrin, plumbagin, quercetin, quercetin-3-*O*-β-D-glucoside, quercitrin, rosmarinic acid, rutin, and silibinin were purchased from Sigma-Aldrich (St. Louis, MO, USA). 5,7,8-Trihydroxyflavone, 5-deoxykaempferol, acacetin, apigenin, apigetrin, β-escin, chrysoeriol, chrysophanol, coumarin, cyanidin, delphinidin, diosmetin, diosmin, ellagic acid, eriocitrin, eriodictyol, eriodictyol-7-*O*-glucoside, eupatorin, ferulic acid, fisetin, flavanone, galangin, gallic acid, genkwanin, gentisic acid, guaiazulene, herniarin, hesperidin, homoeriodictyol, homoorientin, homovanillic acid, hyoscyamine, isorhamnetin, isorhamnetin-3-*O*-glucoside, isorhamnetin-3-*O*-rutinoside, isorhoifolin, juglone, kaempferide, kaempferol, kaempferol-3-*O*-rutinoside, kaempferol-7-*O*-glucoside, kaempferol-7-*O*-neohesperidoside, linarin, liquiritigenin, lutein, luteolin, luteolin tetramethylether, luteolin-3’,7-di-*O*-glucoside, luteolin-4’-*O*-glucoside, luteolin-7-*O*-glucoside, malvidin, mangiferin, maritimein, myricetin, myricitrin, myrtillin, naringenin-7-*O*-glucoside, narirutin, oleuropein, orientin, pelargonidin, pelargonin, phloroglucinol, pyrogallol, quercetin-3-*O*-(-6-acetylglucoside), quercetagetin, quercetin-3,3’,4’,7-tetramethylether, quercetin-3,4’-dimethylether, quercetin-3-methylether, quercetin-3-*O*-glucuronide, rhoifolin, robinin, saponarin, scopolamine, sennoside A, sennoside B, sulfuretin, tectochrysin, tiliroside, verbascoside, and vitexin were obtained from Extrasynthese (Genay, France). Chlorogenic acid was acquired from PhytoLab (Vestenbergsgreuth, Germany). Cinnamic acid was purchased from Biopurify (Chengdug, China). Swertiamarin was obtained from ChemFaces (Wuhan, China). 5,8-Dihydroxy-1,4-naphtoquinone and caffeine were supplied from Fluka (Buchs, Switzerland), vanillin was obtained from Vaz Pereira (Santarém, Portugal), and vicenin-2 from Honeywell (Charlotte, NC, USA).

Roswell Park Memorial Institute (RPMI) 1640 Medium, fetal bovine serum, penicillin/streptomycin solution (penicillin 5000 units/mL and streptomycin 5000 µg/mL), trypsin–EDTA (0.25%), Qubit^®^ RNA IQ assay kit and Qubit^®^ RNA HS assay kit and the SuperScript™ IV VILO™ MasterMix were obtained from Invitrogen (Grand Island, NE, USA). Dimethyl sulfoxide (DMSO) was acquired from Fisher Chemical (Loughborough, UK). Isopropanol was obtained from Merck (Darmstadt, Germany). Dichlorodihydrofluorescein diacetate (DCFH-DA), 3-(4,5-dimethylthiazol-2-yl)-2,5-diphenyltetrazolium bromide (MTT), RNAzol^®^, chloroform, isopropanol, diethyl pyrocarbonate (DEPC), KAPA SYBR^®^ FAST qPCR Kit Master Mix (2X) Universal, protease inhibitor cocktail, sodium dodecylsulfate (SDS), Trizma^®^ hydrochloride, sodium deoxycholate, sodium chloride, potassium phosphate monobasic, potassium chloride, sodium phosphate dibasic, sodium bicarbonate, D-glucose and Triton X-100 were purchased from Sigma-Aldrich (St. Louis, MO, USA). Formaldehyde was from Bio-Optica (Milan, Italy). The ELISA MAX™ Deluxe Set Human IL-6, ELISA MAX™ Deluxe Set Human TNF-α, and ELISA MAX™ Deluxe Set Human IL-1β were supplied by BioLegend (San Diego, CA, USA), while the Caspase-Glo^®^ 1 Inflammasome Assay kit was obtained from Promega Corporation (Madison, WI, USA).

### Cell culture conditions

THP-1 monocytes (American Type Culture Collection, LGC Standards S.L.U., Spain) were maintained in RPMI 1640 medium (p5-p20), containing 10% FBS and 1% penicillin/streptomycin and HEPES at 25 mM. THP-1 Lucia™ NF-κB monocytes (Invivogen, San Diego, CA, USA) were maintained in the same medium containing 100 μg/ml Normocin™ and a selective antibiotic, Zeocin™, was added every other passage at 100 μg/ml, as recommended by the supplier (p2-p8). Both cell lines were kept at 37 ºC with 5% CO_2_.

### MTT reduction assay

THP-1 monocytes were seeded at a density of 6 × 10^4^ cells/well. PMA at 50 nM was added as a differentiation agent into macrophages. After 24 h, this medium was discarded and replaced by fresh PMA-free medium for another 24 h period, after which the differentiated macrophages were incubated with the compounds of interest. 24 h later, the wells were aspirated, and the medium replaced by MTT at 0.5 mg/mL and incubated for 2 h. At the end of this period, the solution was discarded and the formazan crystals in the well were dissolved in 200 µL of a 3:1 DMSO:isopropanol solution. The absorbance was read at 560 nm in a Thermo Scientific™ Multiskan™ GO microplate reader.

### NF-κB luciferase assay

For the evaluation of the NF-κB activation status, a luciferase-based assay was used. This method has been described before [11]. THP-1 Lucia™ NF-κB monocytes were seeded on 96-well plates, differentiated into macrophages as described above for non-transfected THP-1 monocytes, and incubated with the selected compounds. After 2 h, LPS from *E. coli* O111:B4 was added to each well at a final concentration of 1 µg/mL, to induce polarization into M1 pro-inflammatory macrophages. 22 h later, 20 µL of supernatant were collected from each well and transferred to a white 96-well plate. Then, as indicated by the supplier, 50 µL of QUANTI-Luc™ assay solution were added to each well, the plate was shaken, and luminescence was immediately read in a Cytation™ 3 (BioTek) microplate reader.

### Cytokine analysis by ELISA

THP-1 macrophages were obtained as described before on 96-well plates. After exposure to LPS on the presence of the molecules under study, supernatants were collected, and the cells were lysed in RIPA buffer containing 1% protease inhibitor cocktail for 30 min on ice. The concentration of tumor necrosis factor-α (TNF-α), interleukin-6 (IL-6) and interleukin-1β (IL-1β) in the supernatants was determined by ELISA, following the instructions from the manufacturer of a specific kit for each of the pro-inflammatory cytokines under analysis (BioLegend Inc.; San Diego, CA, USA).

### Detection of Reactive Oxygen Species (ROS) production

THP-1 monocytes were seeded at a density of 6 × 10^4^ cells/well on black-bottomed 96-well plates and the differentiation was carried out as mentioned before. The resulting macrophages were incubated with the compounds of interest and, 2 h later, LPS from *E. coli* was added to each well at a final concentration of 1 µg/mL. Cells were carefully washed with HBSS after 22 h and incubated with the fluorescent probe DCFH-DA at 25 µM for 30 min, in HBSS. Finally, fluorescence at 490/520 nm was read in a Cytation™ 3 (BioTek) microplate reader.

### Caspase-Glo® 1 inflammasome assay

Cells were plated and incubated as above described for the NF-κB luciferase assay, yet directly on white 96-well plates. After the differentiation process, the cells were incubated with the molecules of interest and, 2 h later, LPS was added at a final concentration of 1 µg/mL and incubated for 90 min. Caspase-Glo^®^ 1 Reagent was added to each well, in the absence and presence of the selective caspase-1 inhibitor Ac-YVAD-CHO, according to the instructions provided by the manufacturer. After a period of 60 min of incubation at room temperature, luminescence was read on a Cytation™ 3 (BioTek) microplate reader.

### RNA extraction and quantification, conversion to cDNA and RT-qPCR

Cells were seeded at 4.8 × 10^5^ cells/well in 12-well plates, left at 37 ºC for 24 h and then incubated with the molecules selected in previous assays. After 2 h, LPS from *E. coli* at 1 µg/mL was added. The plates were then left in the incubator for a period of 16 h. Samples were collected in 500 µL of PureZOL RNA isolation reagent. The lysate was kept at room temperature for 5 min and then RNA extraction was performed by phase separation by adding 100 µL of chloroform, shaking, incubating for 5 min and centrifuging at 12,000 *g* for 15 min at 4ºC. The aqueous phase was collected and 250 µL of isopropyl alcohol were added, following 5 min of incubation and new centrifugation at 12,000 *g* for 10 min at 4 ºC. The supernatant was then discarded, and the pellet was washed with 75% ethanol, vortexed, centrifuged at 7500 *g* for 5 min at 4 ºC, air dried and resuspended in DEPC-treated water. The RNA in the solution was quantified resorting to the Qubit^®^ RNA IQ assay kit. Sample integrity was then evaluated with the Qubit^®^ RNA IQ assay kit, and, if in appropriate conditions, 1 µg was converted to cDNA using the SuperScript™ IV VILO™ MasterMix. Primers for target genes were designed on Primer BLAST (NCBI, Bethesda, MD, USA) and synthesized by Thermo Fisher (Waltham, MA, USA). The respective nucleotide sequences are presented on Table 1. RT-qPCR cycles consisted in 3 min at 95 ºC, 40 cycles of 95 ºC for 3 s, gene-specific temperature for 20 s and 20 s at 72 ºC and took place in a qTOWER3 G (Analytik Jena AG, Germany), using the KAPA SYBR^®^ FAST qPCR Kit Master Mix (2X) Universal mastermix. Data were analyzed resorting to the qPCRsoft 4.0 software. Product specificity was verified with melting curves. *Gapdh* was selected as reference genes for expression normalization.

**Table 1:** Genes studied by qPCR, NCBI accession numbers and respective primers, annealing temperatures and amplification product size

Gene	Accession number	Primers	Annealing Temperature (ºC)	Amplicon length (bp)
*gapdh*	NM_002046.6	F: AGGTCGGAGTCAACGGATTT	60	157
		R: TGGAATTTGCCATGGGTGGA		
*hspa5*	NM_005347.4	F: ACTCCTGAAGGGGAACGTCT	59.5	161
		R: TTTTCAACCACCTTGAACGGC		
*ddit3*	NM_001195053.1	F: AAGTCTAAGGCACTGAGCGT	59	93
		R: TTGAACACTCTCTCCTCAGGT		
*hsp90β1*	NM_003299.2	F: GCTCTATGTGCGCCGTGTAT	60.5	91
		R: ATCTGAGTCCACCACACCCTT		
*atf4*	NM_001675.4	F: ACAACAGCAAGGAGGATGCC	60	135
		R: CCAACGTGGTCAGAAGGTCA		
*edem1*	NM_014674.2	F: GCGGGGACCCTTCAAATCT	60	117
		R: CGGCTTTCTGGAACTCGGAT		

### Statistical analysis

Statistical analysis was performed in the GraphPad Prism 8 software. The unpaired Student’s t-test was used to compare single treatments with control groups. Values of *p* < 0.05 were considered statistically significant. The Grubbs’ test was resorted to in order to detect outliers.

## Results and discussion

### Selection of nontoxic molecules and their effect upon NF-κB signaling pathway

**Fig. 1:**
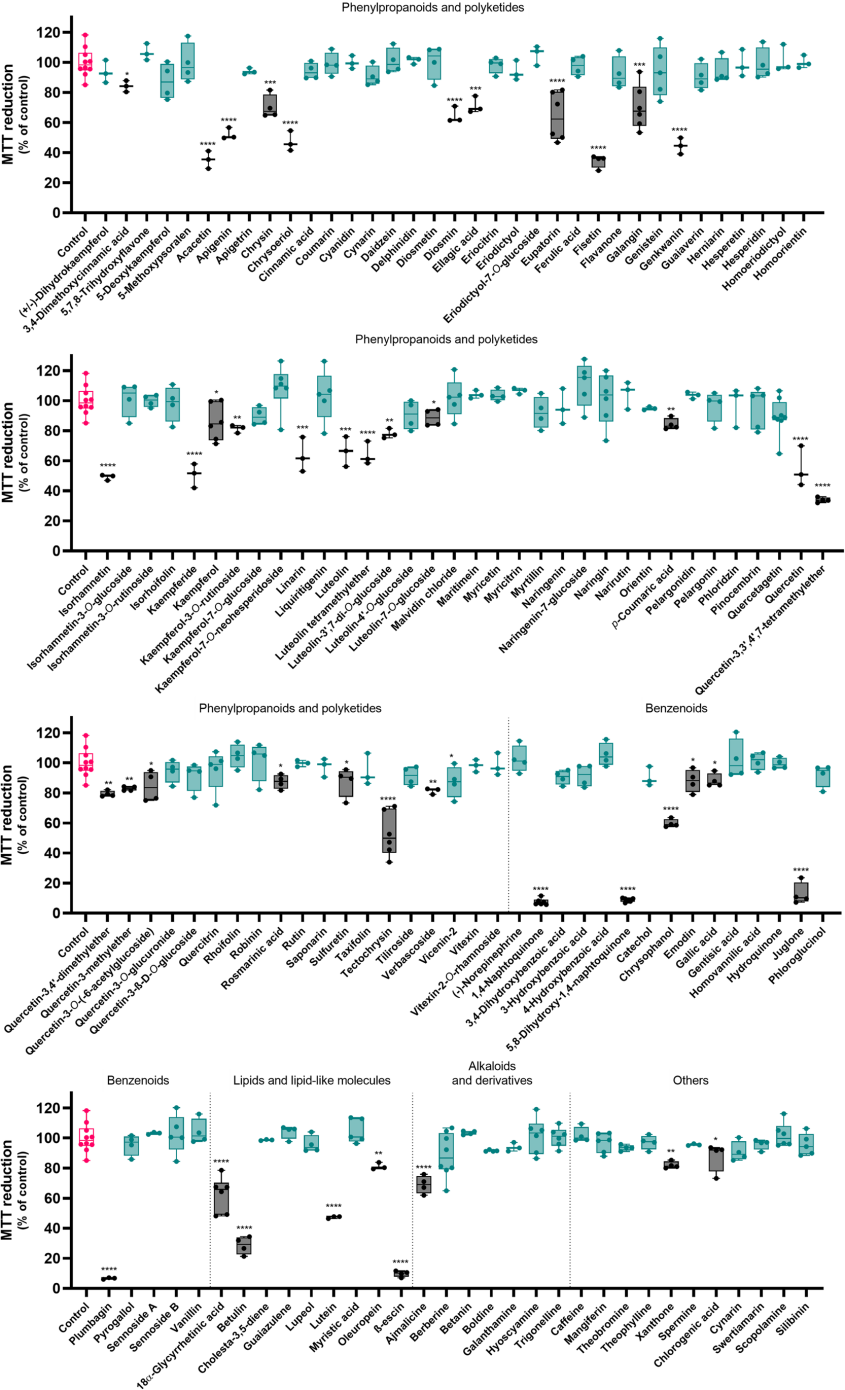
Impact of each molecule upon cell viability of THP-1 macrophages after 24 h, as determined by the MTT reduction assay. All molecules were tested at 50 µM. Results are expressed as percentage of the control and correspond to the mean of at least three independent experiments, individually performed in triplicate. The control group is presented in pink, while the positive control (LPS) is presented in purple. The molecules under study are represented in green whenever active, and in black when inactive. *p < 0.05, **p < 0.01, ***p < 0.001, ****p < 0.0001

In the literature, THP-1 macrophages have been extensively used as a cell model of monocyte/macrophage signaling pathways [5]. The first step of our work was to assess the impact of the molecules under study upon cell viability of THP-1 macrophages (Fig. 1), as a strategy to identify and remove potentially toxic molecules early in the pipeline.

Due to the large number of molecules, all of them were tested at the single concentration of 50 µM. Among all compounds, 46 had a negative impact in cell viability, reason for which they were removed from the study. The remaining 88 molecules were kept and proceeded to subsequent studies.

**Fig. 2:**
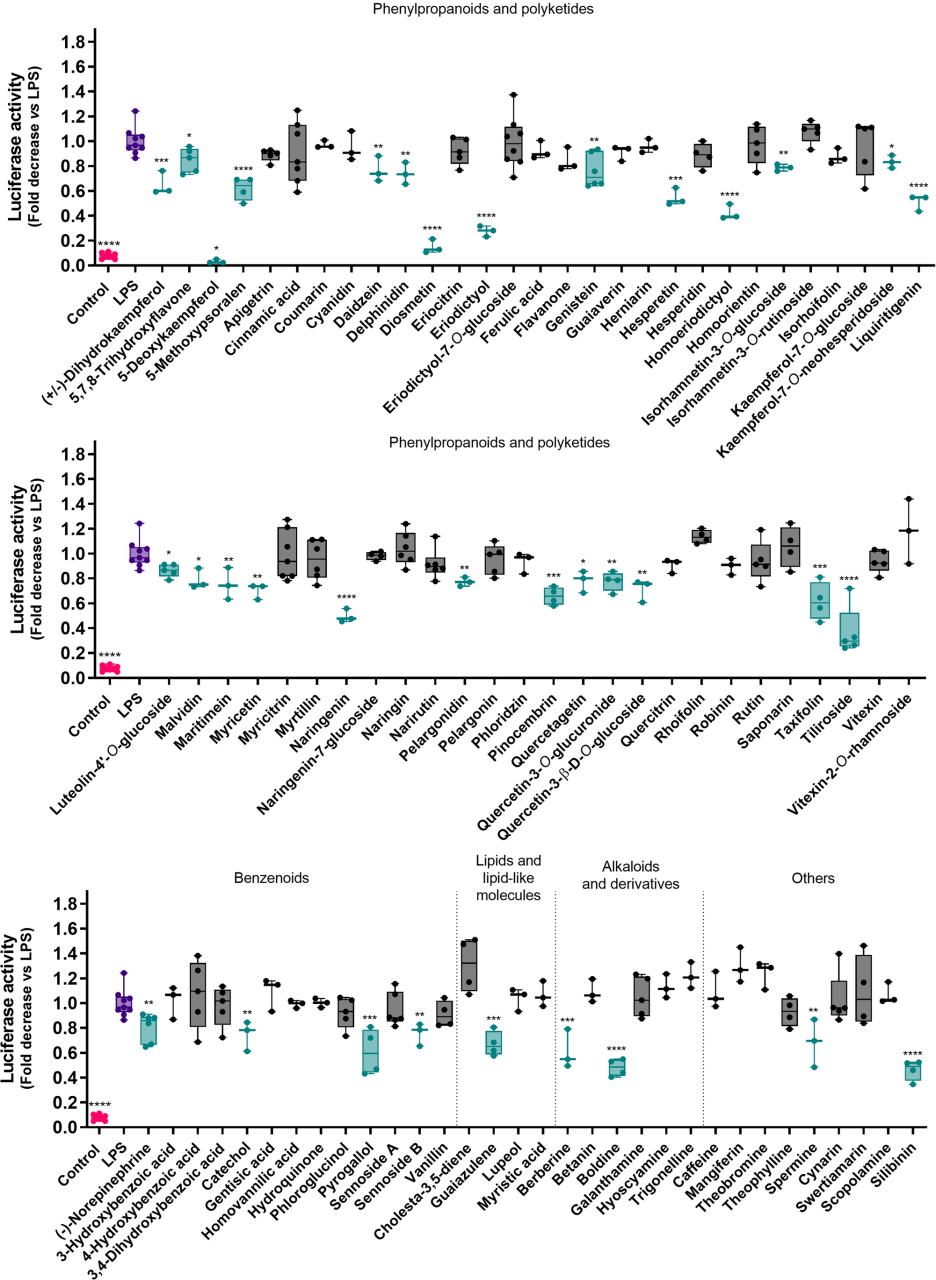
Effect of nontoxic natural products upon NF-κB gene expression on THP-1 Lucia™ NF-κB cells after 24 h, as determined by the QUANTI-Luc™ luciferase activity assay. The control group is presented in pink, while the positive control (LPS) is presented in purple. The molecules under study are represented in green whenever active, and in black when inactive. *p < 0.05, **p < 0.01, ***p < 0.001, ****p < 0.0001

Nontoxic molecules arising from the experiment described above were tested for their ability to decrease NF-κB signaling in LPS-activated macrophages, using a luciferase-based activity assay. NF-κB is a pivotal inflammatory mediator, inducing the expression of multiple downstream pro-inflammatory genes [33]. From the set of compounds tested herein (Fig. 2), around 40% (35 molecules) were able to reduce luciferase activity/NF-κB signaling in a significant manner. The remaining molecules were dropped from the pipeline.

Our set of molecules of interest to study subsequently in mechanistic studies was scaled down to 35 molecules displaying the inhibition of NF-κB signaling. Among these molecules, the ones displaying the most striking activity were 5-deoxykaempferol (over 95% of inhibition) eriodictyol (about 70% of inhibition), homoeriodictyol (around 60%), and diosmetin (over 80%). Additionally, there are several molecules that display the ability to inhibit LPS-induced NF-κB activation at a rate of 50% or more, as can be observed in Fig. 2.

### Impact of anti-inflammatory molecules on inflammation-triggered ROS production

**Fig. 3:**
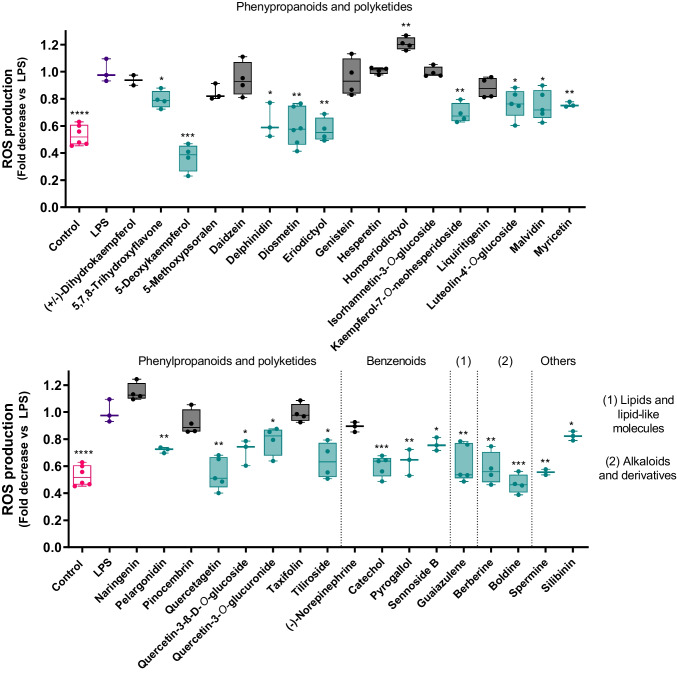
Effect of anti-inflammatory molecules on ROS production on THP-1 macrophages after 24 h of incubation, as determined with the fluorescent probe DCFH-DA. Results express the relative change against the positive control and represent the mean of at least three independent experiments. The control group is presented in pink, while the positive control (LPS) is presented in purple. The molecules under study are represented in green whenever active, and in black when inactive. *p < 0.05, **p < 0.01, ***p < 0.001, ****p < 0.0001

We evaluated whether our active compounds (i.e., those that inhibited NF-κB signaling in the previous experiment), could inhibit ROS overproduction in a context of inflammatory phenotype, namely M1 macrophages. The results narrowed down our group of target compounds from 35 to 22 (Fig. 3) that inhibited ROS overproduction in LPS-activated macrophages, showing that several molecules mitigated the LPS-induced increased generation of ROS. The molecules that failed to exert an inhibitory effect were excluded from the study. In this scope, the strongest activities were displayed by the flavonoids 5-deoxykaempferol, diosmetin, eriodictyol, quercetagetin and the alkaloids berberine and boldine.

### Effect of selected molecules upon proinflammatory cytokine secretion and impact upon inflammasome formation

**Fig. 4:**
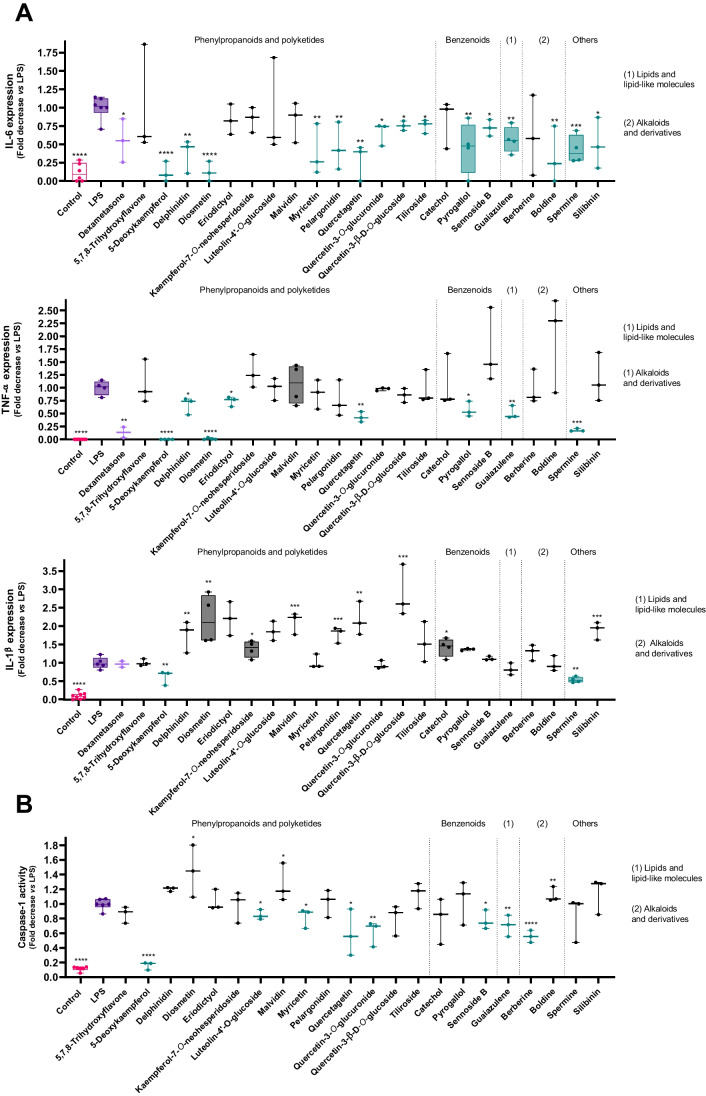
**A**: Influence of active molecules on the expression of IL-6, TNF-α and IL-1β, evaluated by ELISA. Results presented as mean ± standard deviation of the mean of at least three independent experiments, each conducted in triplicate. **B**: Effect of selected molecules on LPS-induced NLRP3 inflammasome activation, as determined by caspase-1 activation. Each column represents the mean ± standard error of the mean of at least three independent experiments, individually performed in duplicate. The control group is presented in pink, while the positive control (LPS) is presented in purple. The molecules under study are represented in green whenever active, and in black when inactive. *p < 0.05, **p < 0.01, ***p < 0.001, ****p < 0.0001

Most the 22 tested molecules, displayed a capacity to significantly inhibit the expression of the IL-6 cytokine, as shown in Fig. 4, with particular emphasis on molecules like 5-deoxykaempferol, delphinidin, diosmetin and myricetin. The active molecules total 15 that resulted in the inhibition of IL-6 production in this incubation period, at a concentration of 50 µM.

Furthermore, eight compounds, mostly of phenolic nature, successfully inhibited the LPS-induced expression of TNF-α (Fig. 4). 5-Deoxykaempferol and diosmetin stand out by their ability to reduce the levels of this pro-inflammatory cytokine to levels equivalent to those of the control group. At the same concentration of the molecules under study, dexamethasone was employed as a positive control, inhibiting the release of both pro-inflammatory cytokines. Most of the selected compounds were able to inhibit LPS-mediated IL-6 upregulation, while this effect was less frequent at the level of the TNF-α production. In what concerns IL-1β, only two molecules under study result in its inhibition.

Among the molecules that build our library at this point, eight were effective at significantly reducing the LPS-induced activation of caspase-1 under our experimental conditions, and thus presumed inhibitors of NLRP3 inflammasome activation (Fig. 4). The molecules that did not prevent caspase-1 activation were removed from the study.

### Effect of anti-inflammatory molecules on UPR-related gene expression

**Fig. 5:**
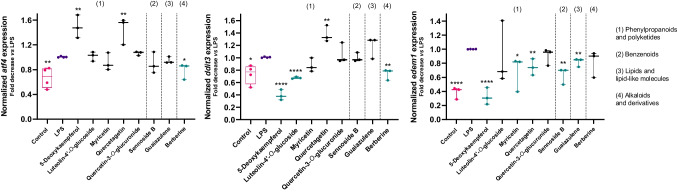
Effect of LPS on the expression of the UPR-related genes coding for *atf4*, *ddit3* and *edem1*, as determined by qPCR. *Gapdh* was the selected reference gene. Results represent the mean ± standard error of the mean of three independent experiments, individually performed in duplicate. The control group is presented in pink, while the positive control (LPS) is presented in purple. The molecules under study are represented in green whenever active, and in black when inactive. *p < 0.05, **p < 0.01, ***p < 0.001, ****p < 0.0001

Incubation with LPS at 1 µg/mL for 16 h results in the upregulation UPR-related genes *atf4*, *ddit3* and *edem1* (Fig. 5). However, the same conditions did not result in significant increases in the reticular chaperones *hspa5* and *hsp90β1* (data not shown). As it was mentioned before, activation of the UPR is characterized by the increased expression of multiple target genes, including *atf4*, *ddit3* and *edem1*, and thus this experimental model faithfully encompasses traits of inflammation (Fig. 4) and UPR signaling (Fig. 5).

The molecules displaying ability to downregulate cytokine release or NLRP3 inflammasome activation, as determined by the previous assay, were tested for their capacity to reduce the impact of LPS-induced UPR signaling on THP-1 macrophages. The results indicate that berberine is the only among these molecules that can significantly inhibit *atf4* expression under the stated experimental conditions (Fig. 5). Furthermore, this molecule also inhibited the expression of the other tested transcription factor, *ddit3*. 5-Deoxykaempferol and guaiazulene have resulted in a significant inhibition of both *ddit3* and *edem1* genes. Luteolin-4’-*O*-glucoside was the only compound revealing the ability to inhibit only the expression of *ddit3*. Finally, myricetin, quercetagetin, and sennoside B inhibited the LPS-induced increase in the expression of *edem1*. According to these results, we have attributed a dual anti-inflammatory and anti-ER stress activity to eight molecules among our chemical library.

## Discussion

Our library initially comprised 134 molecules, and their detailed physico-chemical properties have been published elsewhere by us [10]. The library comprised 65.9% of phenylpropanoids/polyketides, 14.4% of benzenoids and 6% of lipids and lipid-like molecules. Within phenylpropanoids/polyketides, the most prevalent subclass was flavonoids, followed by cinnamic acids and coumarins, all of which are widespread in vegetable species present in human diet.

**Fig. 6:**
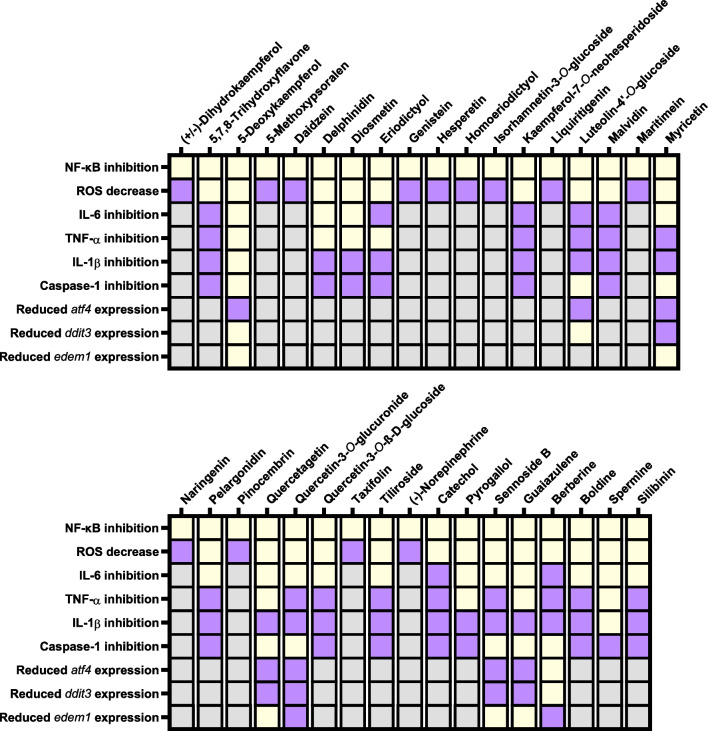
Summary of the obtained results concerning every NF-κB inhibitor discussed herein. Black cells stand for not applicable conditions, yellow cells represent an active molecule and purple cells represent a negative result

Among these molecules we have identified 35 molecules that inhibit LPS-induced inflammation at the concentration of 50 µM, as proved by their effect upon NF-κB activation. These active molecules were then tested in a battery of subsequent assays to characterize this activity, and the obtained results are summarized and Fig. 6. The ability to counter or inhibit NF-κB signaling is an important indicator of anti-inflammatory activity. NF-κB is rendered inactive by negative regulation by IκBα, which is bound to the transcription factor under homeostatic conditions. Whenever a cytokine or pattern recognition receptor recognizes a ligand, the activation of a signaling cascade takes place, releasing NF-κB and allowing its activity as a transcription factor, and thus inducing inflammatory gene expression [12]. For this reason, we excluded molecules that failed to inhibit NF-κB signaling from our library.

The relationship between oxidative stress and inflammation is a two-way street. One does not occur without the other, and the increase of one enhances the other [43]. It is known that NF-κB signaling regulates ROS generation, and, conversely, that ROS can positively or negatively impact NF-κB signaling [32, 35]. ROS overproduction leads to the phosphorylation of the I*κ*B*α*, leading to its degradation by the proteasome, resulting in NF-κB activation [61]. On the other hand, ROS production is inherent to protein folding, and ER stress implicates oxidative stress [60]. Inhibiting ROS overproduction can therefore be of interest to ameliorate inflammatory processes, and for that reason this was the ensuing exclusion criterion. Molecules that failed to reduce LPS-induce ROS generation were excluded of further experiments. Notably, the past few years have witnessed the discovery of an additional crosstalk between oxidative stress and ER stress. The generation of ROS is inherent to protein folding [44] and, on the other hand, increased levels of ROS result in ionic calcium influx into the cytosol, triggering ER stress and further ROS generation [9, 60]. Accordingly, the molecules identified herein as capable of countering ROS overproduction can be of interest when trying to mitigate ER stress. Once again, 5-deoxykaempferol and eriodictyol exhibit a strong biological activity. However, notably, homoeriodictyol, which was a strong inhibitor of NF-κB signaling, did not result in ameliorated ROS generation. This molecule, along with others with the same pattern of activity, did not follow through to the next steps in this work, since the selection of the molecules that followed through was based on a significant inhibitory activity.

The next step in this study was the evaluation of the impact of active molecules upon LPS-induced pro-inflammatory cytokine expression. Inflammatory signaling is largely regulated by cytokines. Interleukin-6 (IL-6), tumour necrosis factor (TNFα) and interleukin-1β (IL-1β) are key pro-inflammatory cytokines that signal via the type I cytokine receptors. All of these cytokines are abundantly secreted by M1 macrophages [49]. For this reason, we were interested in studying the ability of the molecules under study to negatively modulate these cytokines in macrophages. This resulted in the identification of a few molecules devoid of activity: 5,7,8-trihydroxyflavone, berberine, catechol, kaempferol-7-*O*-neohesperidoside, luteolin-4’-*O*-glucoside and malvidin did not reduce the secretion of the analyzed pro-inflammatory cytokines. We hypothesize the NF-κB inhibition is insufficient with this concentration of the molecule, and/or in the chosen experimental period, to allow the observation of the reduced cytokine expression that was expected, as hinted by a generally weaker activity of these compounds in the NF-κB activity and ROS production assays presented above. Boldine, myricetin, pelargonidin, quercetin-3-β-D-*O*-glucoside, quercetin-3-*O*-glucuronide, sennoside B, silibinin, and tiliroside exclusively reduced the production of IL-6, while eriodictyol lead to a mitigated secretion of TNF-α only. Finally, delphinidin, diosmetin, guaiazulene, pyrogallol, and quercetagetin inhibited the release of both IL-6 and TNF-α.

In our experimental model, the molecules tested were the least effective against the release of IL-1β. Only two molecules were effective against the production of all of the three major inflammatory cytokines, and those were 5-deoxykaempferol and spermine. Notably, the reference anti-inflammatory compound dexamethasone also failed to exert this effect under the stated experimental conditions, even though this molecule is employed in therapeutics, implying that IL-1β *per se* is not a sufficiently robust parameter to assess anti-inflammatory activity. Furthermore, the same phenomenon has been described before in mice [47].

Innate immune cells contain the intracellular sensor NOD-, LRR- and pyrin domain-containing protein 3 (NLRP3), that detects danger signals (pathogens, environmental or endogenous), acting as a sensor of cellular stress, leading to the assembly and activation of the NLRP3 inflammasome and subsequent processing of procaspase-1 into catalytically active caspase-1, which, in turn, results in the activation and release of cytokines IL-1β and IL-18, and in the occurrence of pyroptosis [46]. There are reports linking the activation of all three major signaling branches of the UPR to inflammasome activation, mainly IRE1 and PERK. Briefly, UPR signaling promotes NF-κB signaling, increasing the expression of IL-1β and NLRP3. Additionally, UPR activation promotes ROS production and NLRP3 association to the mitochondria, leading to leakage of mitochondrial contents that, in turn, activate NLRP3 [62]. A disturbance in Ca^2+^ levels is also reported to be linked to NLRP3 activation, being that it can occur both upstream or downstream of this event [46]. Taking this information into account, we have determined the potential of the molecules under study to inhibit caspase-1 activity, inferring their potential to inhibit inflammasome activation. A statistically significant activity in this assay was used as a selection criterion to transport molecules into the following assays, that aimed to unveil the relationship between the observed anti-inflammatory activity and ER stress amelioration.

Inflammatory and ER stress signaling share the trait of being activated in order to protect cellular function. There are multiple signaling of crosstalk between these two molecular machineries. In fact, NF-κB activation may result from the activation of any of the three major signaling branches of the UPR, whereas inflammatory signaling results in UPR activation. Furthermore, both signaling pathways can be deleterious when intensely and/or chronically induced [6].

The gene *atf4* encodes the activating transcription factor 4 (ATF4), a transcription factor that is translated in response to the activation of the PERK branch of the UPR. Activation of PERK causes increased phosphorylation levels of the eIF2α, leading to an overall halt in protein synthesis. However, eIF2α phosphorylation promotes the synthesis of a few target proteins, such as the ATF4. In turn, ATF4 induces the expression of specific genes, including *ddit3*, the gene that codes for the CHOP transcription factor [1]. The latter is involved in ER stress-associated regulated cell death mechanisms when the activation of the UPR fails to restore proteostasis [21].

The ER degradation-enhancing alpha-mannosidase-like protein 1 (EDEM1), the enzyme that corresponds to the *edem1* gene, participates in endoplasmic reticulum-associated degradation (ERAD) of misfolded glycoproteins, by removing them of the calnexin cycle. Enhancement of ERAD is known to be a downstream event of the activation of the ATF6 signaling branch of the UPR [2, 17].

As all of these molecules inhibited inflammatory signaling in previous assays, we can confirm the dual activity of 5-deoxykaempferol, berberine, guaiazulene, luteolin-4’-*O*-glucoside, myricetin, quercetagetin and sennoside B. Even though at different levels, these natural products can significantly mitigate inflammatory and UPR signaling. The genes *atf4* and *ddit3* are more associated to the deleterious effects of ER stress signaling, since, as mentioned before, ATF4 induces the expression of CHOP, that, in turn, is involved in ER stress-induced regulated cell death. For this reason, we hypothesize that berberine is the most promising molecule within our group, given that it was the only that significantly inhibited the expression of both genes in our experimental model. However, *atf4* can also be associated to the adaptive effects of UPR activation, unlike *ddit3*. Thus, the effects of luteolin-4’-*O*-glucoside should also be considered, even though it only inhibited the expression of *ddit3* in a significant manner.

Increased EDEM1 expression enhances ERAD, promoting proteasome-mediated degradation of toxic misfolded proteins. Loss of mannoses in these peptides indicates that they have remained in the calnexin cycle for too long to endure several failed attempts at correct folding, and they should be forwarded for degradation [37]. Diminished *edem1* expression might indicate a reduced presence of toxic misfolded proteins in the lumen of the ER, and, for this reason, 5-deoxykaempferol, guaiazulene, myricetin, quercetagetin, and sennoside B have shown to preserve homeostatic conditions in the organelle. The activity of 5-deoxykaempferol is worth highlighting, since it was among the most potent molecules in every other assay that was performed.

To our knowledge, this is the first report of 5-deoxykaempferol as an inhibitor of inflammasome or caspase-1 activation, even though its anti-inflammatory potential has already been described in a murine macrophage model. In RAW264.7 cells, this molecule inhibited NO production, TNF-α and IL-1β mRNA expression, as well as iNOS and COX-2 protein expression, at the same concentration that was employed in this study (50 µM) and lower concentrations [58]. In concentrations up to 75 µM, albeit with a different incubation period, berberine inhibited inflammasome activation and IL-1β secretion in THP-1 macrophages [24]. A similar effect has been observed in BV2 [22] and AML12 cells [34]. Notably, NLRP3 inhibition and IL-1β downregulation by this compound have also been observed in vivo [50]. In Caco-2 cells (intestinal epithelial cells), berberine seems to prevent tunicamycin- or proinflammatory cytokine-induced ER stress. Under these conditions, berberine reduced GRP78 expression and XBP-1 splicing, and downregulated JNK, caspase-12 and caspase-3, successfully preventing apoptosis. For these reasons, the authors hypothesise that berberine can be useful against the pathogenesis of the inflammatory bowel disease [18]. Guaiazulene bioactivity in the scope of inflammation, to the extent of our knowledge, has not been characterized so far. Luteolin-4’-*O*-glucoside has reduced the levels of IL-1*β* and TNF-*α* and displayed the ability to reduced paw edema in mice [31]. However, there are no other reports concerning the anti-inflammatory potential of this molecule, namely at the level of inflammasome activation. Myricetin was reported to inhibit NF-κB signaling in RAW264.7 macrophages (25–100 µM), as well as in mice. Interestingly, this effect results from suppressed degradation of the IκBα [8]. Besides inhibiting NF-κB signaling, myricetin is described as a NLRP3 inflammasome activation inhibitor (25–75 µM), by inducing NLRP3 ubiquitination and reducing ASC ubiquitination, therefore blocking inflammasome assembly, in primary peritoneal macrophages (PMs) [7]. To the extent of our knowledge, there are no other reports of quercetagetin as an inhibitor of inflammasome activation. However, its anti-inflammatory potential has been evaluated before, albeit in cell models different than those we employed here. In H9c2 rat myoblasts, quercetagetin (10 µM) decreased COX-2 expression and decreased IκBα degradation [16]. Furthermore, in HaCaT human keratinocytes, quercetagetin (12.5–50 µM) inhibited the production of the thymus and activation-regulated chemokine (TARC) and macrophage-derived chemokine (MDC) [25]. Quercetin-3-*O*-glucuronide inhibited ROS overproduction in human umbilical vein endothelial cells (HUVECs), as well as IL-6 and TNF-α protein levels, at concentrations from 1 µM [15]. In LPS-insulted RAW 264.7 macrophages, at concentrations from 50 µM, quercetin-3-*O*-glucuronide inhibited NO and PGE_2_ production, as well as iNOS and COX-2 expressions at the protein level, while inhibiting JNK activation from 10 µM [38]. Furthermore, this natural product has displayed its anti-inflammatory activity in vivo, inhibiting ear edema in mice [13]. Recently, sennoside B was identified as a strong inhibitor of the activity of TNF-α on a competitive binding assay based on analytical size exclusion chromatography, even though the effect at the gene level expression is not reported [39].

The literature conveys no reports of the activity on 5-deoxykaempferol against ER stress besides our previous study, in which we describe this molecule’s ability to counter thapsigargin-induced ER stress [10]. As so, we hypothesize that the protective effect of this molecule against ER stress may be involved in the anti-inflammatory effect here reported. In HepG2 cells, berberine has demonstrated to inhibit ER stress, as evidenced by the decreased levels of phosphorylation PERK and eIF2α in tunicamycin-insulted cells after exposure to berberine up to 20 µM [52]. In a mice model, this molecule was effective in reducing ER stress, as observed by the decrease in the expression of BiP, p-PERK and p-eIF2α, that resulted in the inhibition of tau hyperphosphorylation [53]. Berberine was protective against palmitate-induced ER stress in a mouse podocyte cell line (MPC5), reducing the expression of multiple ER stress biomarkers [54]. Myricetin triggered the efflux of Ca^2+^ into the cytosol, while increasing the expression of UPR markers in concentrations from 20 µM, in JAR and JEG-3 cells (both from placental choriocarcinomas) [56]. This concurs with results observed on an ovarian cancer cell line (SKOV3), in which an increase of BiP expression was observed upon treatment with myricetin (40 µg/mL) [55]. However, this molecule has also displayed protective potential against ER stress before (20 µM), protecting rat primary pancreatic β-cells from high glucose-induced apoptosis and preventing the abnormal expression of UPR biomarkers, such as BiP, p-PERK, p-eIF2α, ATF4 and CHOP [26]. Regarding luteolin-4’-*O*-glucoside, quercetagetin and sennoside B, we find no accounts in the literature of their potential to modulate ER stress.

Known ER stressors, such as thapsigargin and tunicamycin, are known to activate NF-κB, which can result from the activation of any of the three major signaling branches of the UPR. ER stress inhibition proved efficient in reducing inflammation in several in vitro and in vivo models [6]. UPR activation leads to a halt in global protein synthesis rates. The mechanism through which the PERK branch of the UPR can trigger NF-κB lies in this fact, since this halt exerts a crisp effect upon short-lived proteins, as is the case of IκB [6] Nonetheless, it selectively enhances the translation of genes that possess internal ribosome entry sites, such as ATF4. Concurrently, the kinase activity of IRE1 maintains the activity of IKK [48]. Similarly, activation of the ATF6 signaling branch of the UPR has also been observed to induce NF-κB activation and cytokine release, and also enhance cytokine synthesis [36, 45]. Furthermore, protein folding is carried out by the ER. This process is responsible for up to 25% of the generation of ROS within a cell, as the organelle needs to maintain an oxidizing environment for the formation of disulfide bonds.

The ER is the major regulator of Ca^2+^ signaling. Abnormal ROS production and abnormal Ca^2+^ levels generate a positive feedback loop between each other, given that excess ROS can also promote the cellular uptake of Ca^2+^ [59]. Furthermore, Ca^2+^ signaling impacts inflammasome activation. NLRP3 inflammasome activation implies two signaling steps: the first relies on NF-κB signaling and is termed priming; the second leads to assembly and activation of the inflammasome, resulting in caspase-1 activation and maturation and release of IL-1β [46]. Activation can be induced by any NLRP3 agonist, like the efflux of K^+^ or Ca^2+^ ions from the ER, ROS or proteases, resulting in caspase-1 activation and maturation and release of IL-1β [7, 33].

Cellular organelles, namely the ER and mitochondria, participate in NLRP3 inflammasome activation. The influx of Ca^2+^ from the ER stores into the mitochondria leads to the leakage of mitochondrial contents and results in NLRP3 activation. Furthermore, the area between the ER and mitochondria provides a platform for NLRP3 inflammasome assembly, since the inactivated NLRP3 locates mainly in the ER but translocates to this interface upon activation [62]. Even though, in most cases, whether ER stress is a cause or consequence of a particular disease is yet to be defined, there is a consensus in that it can be a therapeutic target. Notably, mitigating ER stress associated to inflammation has proven to result in few undesirable side effects [6].

## Conclusions

**Fig. 7:**
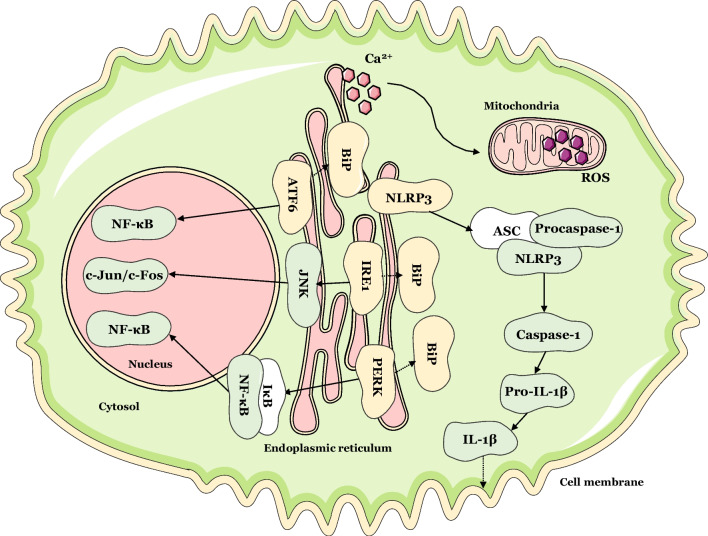
Crosstalk between ER stress and inflammatory signaling

In the present day, a significant part of the most prevalent chronic diseases are of inflammatory nature [49]. On the other hand, ER stress and activation of the UPR is deeply associated with chronic diseases [20, 27, 51]. Conversely, it is increasingly documented that inflammatory pathways are highly linked to UPR activation (Fig. 7) [30]. It has been suggested the foundation of chronic inflammatory disease may be the NLRP3 inflammasome activation triggered by ER stress. The two phenomena are deeply connected, since ER stress may trigger NLRP3 inflammasome activation via oxidative stress, Ca^2+^ signaling and NF-κB activation [29].

For this reason, there is a need for molecules that can display a dual effect against both inflammatory and UPR signaling. This work led to the identification of promising drug candidates against these diseases among a library of small molecules of natural origin, being that 5-deoxykaempferol stood out as the most auspicious molecule.
